# Taste and smell disturbances in patients with chronic oral graft vs. host disease: An observational study

**DOI:** 10.3389/froh.2022.934607

**Published:** 2022-09-09

**Authors:** Marlou Boor, Judith E. Raber-Durlacher, Mette D. Hazenberg, Frederik R. Rozema, Alexa M. G. A. Laheij

**Affiliations:** ^1^Department of Oral Medicine, Academic Centre for Dentistry (ACTA), University of Amsterdam and Vrije Universiteit Amsterdam, Amsterdam, Netherlands; ^2^Department of Oral Maxillofacial Surgery, Amsterdam UMC, University of Amsterdam, Amsterdam, Netherlands; ^3^Department of Hematology Amsterdam UMC, University of Amsterdam, Amsterdam, Netherlands; ^4^Department of Preventive Dentistry, Academic Centre for Dentistry (ACTA), University of Amsterdam and Vrije Universiteit Amsterdam, Amsterdam, Netherlands

**Keywords:** hyposalivation, hypogeusia, quality of life, allogeneic hematopoietic stem cell transplantation (alloHSCT), chronic oral graft-vs.-host disease, taste and smell disturbances

## Abstract

**Background:**

A common complication of allogeneic hematopoietic stem cell transplantation (alloHSCT) is chronic oral graft vs. host disease (cGvHD). Oral cGvHD may present as mucosal lesions, salivary gland dysfunction, and trismus. Moreover, taste and smell ability may be affected, but the prevalence, nature and severity of altered taste and smell function, and their impact on quality of life (QoL) are understudied.

**Aim:**

To identify the prevalence, nature, and severity of taste and smell disturbances, their impact on QoL and to assess whether altered taste/smell ability is associated with oral mucosal cGvHD or hyposalivation.

**Materials and methods:**

AlloHSCT recipients at least 100 days post-HSCT and referred for oral cGvHD-related oral complaints were eligible for participation in this cross-sectional study. Manifestations of oral mucosal cGvHD were scored, the (un)stimulated salivary flow was measured, and objective taste and smell ability was evaluated. Subjective taste and smell alterations, and overall and oral health (OH)-related QoL were assessed.

**Results:**

In total, 45 patients were included, of which objective reduced taste ability (hypogeusia) was identified in 68.9%; 28.9% had reduced smell ability and 11.1% had complete loss of smell. Nevertheless, only 31.1% of patients reported severe taste alterations and 22% reported moderate taste alterations indicating that not all the patients were aware of their altered taste sense. Taste/smell disturbances were not related to oral mucosal cGvHD or hyposalivation. Most alloHSCT recipients reported a decreased OH-related QoL. However, a relation between taste/smell ability and global or OH-related QoL could not be identified.

**Conclusion:**

Taste and smell disturbances are prevalent among alloHSCT recipients. Most patients reported a decreased OH-related QoL, but the specific impact of taste and smell disturbances remains to be elucidated.

## Introduction

Chronic graft vs. host disease (cGvHD) is a common complication of allogeneic hematopoietic stem cell transplantation (alloHSCT) [1, 2]. Patients receive stem cells collected from peripheral blood, bone marrow, or umbilical cord blood from a related or unrelated donor. Immune cells derived from these donor stem cells (the graft) eradicate malignant cells in hematological malignancies, but may also interact with normal host cells. This allo-immune response can affect various organs, usually targeting the skin, eyes, mouth, gastrointestinal tract, liver, lungs, musculoskeletal and genitourinary system, resulting in cGvHD, that may be associated with pain, severe impaired function and poor quality of life (QoL) [1, 2].

The oral cavity is estimated to be involved in 45–83% of patients with cGvHD [1]. Oral cGvHD can develop at any oral or orofacial site and may present as mucosal lichenoid hyperkeratotic changes, ulcerations, redness, sensitivity/pain, mucoceles, salivary gland dysfunction, reduced mouth opening, and taste impairment (dysgeusia or hypogeusia) [1–6].

Human flavor perception is a complex entity that interacts with taste, smell, somatosensory signals (texture and temperature), and psychological elements [7]. Taste buds can distinguish five basic tastes: sweet and umami serve intake of high calorie food and pleasure of eating, bitter warns for unbecoming ingredients, and salt and sour are integrated in the homeostasis of the ionic and osmotic regulation [8]. Studies on altered taste function in the alloHSCT recipients reported a persistent, selective alteration in umami, salty and sweet taste by 47% of patients even years after transplant [4, 9].

Receptors of the olfactory nerve (cranial nerve I) are clustered in the small area in the back of the nasal cavity, facilitating the detection of/and response to odor molecules provided by chewing and swallowing. A heightened sensitivity to odors or a complete loss of smell can hinder nutritional intake by reducing the ability to taste and enjoy eating and drinking [10].

In addition to a reduced or a complete loss of smell, multiple factors could contribute to the development of taste alterations in alloHSCT recipients such as conditioning regimen-related toxicity, damage to taste buds by oral cGvHD-induced inflammation, neurotoxicity involving the cranial nerves VII, XI, and X, modifications of the oral microbiota, infections including dental diseases, poor oral hygiene, medication use, reduced salivary flow, and increased anxiety [11, 12].

Although there is some evidence suggesting taste and smell changes in alloHSCT recipients, the prevalence, and severity of these changes and their relation with oral cGvHD are largely understudied. In addition, impaired taste and smell function may lead to malnutrition and provoke feelings of disappointment and sadness that may have a significant negative impact on patient's global and oral health-related QoL (OH–QoL) [13, 14]. Therefore, the aim of this study is to identify the prevalence, nature, and severity of taste, and smell disturbances in patients visiting our oral GvHD clinic and to examine whether taste and smell disturbances are related to the presence and severity of oral mucosal cGvHD, hyposalivation and global, and OH-QoL.

## Materials and methods

This cross-sectional study was conducted at the Department Oral and Maxillofacial Surgery of the Amsterdam University Medical Center, location AMC between February 2019 and December 2020. The study has been approved by the Institutional Medical Ethics Committee (NL69437.018.19). Written informed consent was received from all the participants. All patient data were anonymized before processing and stored in a secured database (Castor EDC, Amsterdam, The Netherlands).

### Eligibility criteria

Patients who received an alloHSCT for a hematological malignancy at least 100 days ago and were referred because of oral cGvHD-related complaints were eligible for inclusion. In addition, patients had to have either manifestations of oral cGvHD or a history of cGvHD-related oral manifestations. Patients were excluded if they were current smokers, had pre-existing autoimmune disorders (Sjögren syndrome or lichenoid granulomatous disorders), neurodegenerative comorbidity (Parkinson's disease or Alzheimer's disease) or uncontrolled diabetes mellitus.

### Oral examination

The oral cavity was examined clinically in order to verify the presence or absence of oral manifestations of cGvHD. All the oral examinations were performed by an experienced dentist specialized in diagnosing and managing oral complications in patients with cancer (JR–D). Mucosal changes were scored using the NIH oral cGvHD Activity Assessment Tool. This scoring system takes into account the severity and extent of erythema, lichenoid hyperkeratotic changes, ulcerations, and mucoceles with a total score ranging from 0 to 15 points [15]. Patients with scores of 0–2 were considered as having no oral cGvHD, whereas scores of 3–15 were considered indicative for the presence of oral cGvHD [16].

### Questionnaires

Questionnaires assessing the gustatory sense and patient-reported oral GvHD (NIH), the quality of life (EORTC QLQ–C30), oral health-related quality of life (EORTC QLQ–OH15 and OHIP-14) were used.

Taste and smell addendum of the EORTC QLQ–C30 is designed to detect patient-reported changes of the sensitivity and the specificity of smell and taste, specifically with respect to the basic tastes of salt, sweet, sour, and bitter [17]. The items were rated on a 4-point Likert-scale: 1 (not at all), 2 (a little), 3 (quite a bit), and 4 (very much).

The NIH questionnaire records self-reported severity of oral cGvHD symptoms: dryness, pain, and sensitivity of the oral cavity at the worst moment over the past 7 days [15, 18]. These items are scored using a 11-point Likert-scale ranging from 0 (not existing) to 10 (the worst imaginable).

The EORTC QLQ–C30 is a validated global QoL questionnaire designed to be self-administered by patients with cancer [19]. The QLQ–C30 consists of multiple subscales: functional scales, symptom scales, and subscales addressing various symptoms (dyspnea, insomnia, loss of appetite, constipation, diarrhea, and financial impact). All the items are scored using a 4-point Likert scale: 1 (not at all), 2 (a little), 3 (quite a bit), and 4 (very much). Global health status subscale is scored using a 7-point Likert scale, 1 (“very poor”) to 7 (“excellent”) [20].

The EORTC QLQ–OH15 is an addition to the EORTC QLQ–C30 that relates oral problems to OH-related QoL in patients with cancer [21]. The items were categorized in 6 subscales: OH–QoL score (8 items), information scale (2 items), scale regarding dentures (2 items), and three single items (sticky saliva/mouth soreness/sensitivity to food/drink). All the items are graded using a 4-point Likert scale: 1 (not at all), 2 (a little), 3 (quite a bit), and 4 (very much). The minimum score on this questionnaire (excluding the information on denture related questions) is: 11, the maximum score is 44. A higher score indicates a reduced oral health-related quality of life.

The Oral Health Impact Profile (OHIP-14) indicates the social impact of OH-related QoL over the past 30 days [22]. The items of the OHIP are divided into seven dimensions: functional limitation, physical pain, psychological discomfort, physical disability, psychological disability, social disability, and handicaps. All the items are evaluated using a 5-point Likert scale: 1 (never), 2 (hardly ever), 3 (occasionally), 4 (fairly often), to 5 (very often). The minimum score of this questionnaire is: 14, and the maximum score is 70. A higher score indicates a reduced OH-related QoL.

### Sialometry

Whole (un-)stimulated salivary flow rates and the salivary pH-values were assessed. Before the saliva measurements, the participants were requested to refrain from eating, drinking (other than water), and any oral hygiene practices for at least 30 min. Measurements were performed between 9:30 and 11:30 am. The procedure consisted of expectoration of all produced (un-)stimulated saliva, continuously for 5 min, into a pre-weighted plastic tube. During the stimulated salivary flow test, patients received a tasteless paraffine chewing gum to stimulate the salivary glands. Patients were asked not to talk and to swallow during the collection of both samples [23]. Salivary flow rates were determined in grams per minute (g/min). Severe hyposalivation was identified when the unstimulated salivary flow rate was below 0.1 g/min and/or the stimulated salivary flow rate was below 0.5 g/min [24].

### Taste evaluation

The Burghart taste strips test (Medisense, Burghart Messtechnik, Wedel, Germany) evaluated the taste sensitivity of the oral cavity as a whole. The 16 taste strips are impregnated with four different flavors in different concentrations: sweet (0.05, 0.1, 0.2, or 0.4 g/ml sucrose), salty (0.016, 0.04, 0.1, or 0.25 g/ml sodium chloride), sour (0.05, 0.09, 0.165, or 0.3 g/ml citric acid) or bitter (0.0004, 0.0009, 0.0024, and 0.006 g/ml quinine hydrochloride). All the strips were offered in a fixed order to every patient, according to the protocol. The patients were asked to place the strip on the tongue and to close the mouth and choose one of the four answer options (sweet, sour, bitter, and salt). If they did not taste anything, flavorless was reported. Hypogeusia was identified if the overall score was lower than 9 (out of 16) [25].

### Smell evaluation

For testing the olfactory performance of the patients, the validated smell test Sniffin' Sticks (Burghart Messtechnik, Wedel, Germany) was used [26]. This diagnostic screening test allows for differentiating the inability in the detection of odors (anosmia) and a reduced ability to detect odors (hyposmia) from a common smell sense (normosmia). Odor pens containing 12 different all-day aromas were used, for example, lemon, coffee, and leather. Patients were asked to place the pen straight under their nose (at a distance of 2 cm) for 3–4 s. They were offered a card with four answers and had to pick the answer which described the presented odorant the best. Anosmia was identified if the overall score was below 6 (out of 12) and hyposmia if the score was between 6 and 9 (out of 12).

### Statistical analysis

Relations between oral GvHD, taste and smell disorders, salivary flow and QoL were calculated using Fisher–Freeman–Halton exact test, the Mann–Whitney *U*-test and the Kruskal–Wallis test. The IBM SPSS Statistics software package (IBM SPSS Statistics version 27, IBM, Armonk, NY) was used to perform all the data analyses. A *p*-value of <0.05 was considered statistically significant.

## Results

### Patient characteristics

In total, 45 recipients treated with allogeneic HSCT (44.4% women: 55.6% men) were enrolled in this study (Table 1). The mean age of the participants was 53 years (±14.7), the most commonly encountered diagnosis was acute myeloid leukemia (30.8%). Patients received an alloHSCT at least 100 days ago. One patient was transplanted more than 10 years ago, but most patients received an alloHSCT between 1 and 3 years ago. Conditioning regimens and other medications were tailored to the diagnosis and specific patients' needs. At the time of their assessment in this study, patients used on average 11.5 (±5.5) different medications, namely, antiviral, antifungal, antibacterial, and immune suppressant medications. All patients used at least one drug that potentially could have affected their taste [27, 28].

**Table 1:** Patient and treatment characteristics.

**Variables**	***n* (%)**,
	**Mean ±SD**
*Age (years)*	53.27 ± 14.727
**Gender**	
Female	20 (44.4%)
Male	25 (55.6%)
**Diagnosis**	
Acute myeloid leukemia	14 (30.8%)
Myelodysplastic syndrome	7 (15.4%)
Angioimmunoblastic T-cell lymphoma	3 (6.6%)
Mantle cell lymphoma	3 (6.6%)
Acute lymphocytic leukemia	2 (4.4%)
Chronic lymphocytic leukemia	2 (4.4%)
Sickle cell anemia	2 (4.4%)
Multiple myeloma	2 (4.4%)
Non hodgkin lymphoma	2 (4.4%)
Other	8 (17.6%)
**Conditioning regimen**	
Myeloablative	11 (24.4%)
Non-myeloablative	14 (31.1%)
Reduced intensity	20 (44.4%)
**Time since transplantation (years)**	
<1	12 (26.7%)
1–3	19 (42.2%)
3–5	8 (17.8%)
>5	6 (13.3%)
**Stem cell source**	
Peripheral progenitor cell	34 (75.6%)
Bone marrow	11 (24.4%)
Number of medications taken that could potentially affect taste	11.5 (± 5.5)

### Oral cGvHD

All the patients had either manifestations of oral cGvHD at the time of assessment in this study or had a recent history of oral cGvHD manifestations diagnosed and treated in our clinic. At the oral examination performed for this study, 24 patients (53.3%) had manifestations of oral mucosal cGvHD. Lichenoid changes (40%) and erythema (36%) were most commonly present and their extent/severity scored highest at the NIH Activity Assessment scoring instrument in Oral cGvHD Activity Assessment Tool. Ulcerations (11%) and mucoceles (13%) manifested less frequently and were mild-to-moderate in the most patients (Table 2). None of the patients had manifestations of mucosal infections.

**Table 2:** Presence and severity of oral mucosal cGvHD scored by the Oral cGvHD Activity Assessment Tool [15].

	**Not present**	**Mild**	**Moderate**	**Severe**
**Erythema**	29 (64.4%)	10 (22.2%)	1 (2.2%)	5 (11.1%)
**Lichenoid**	27 (60.0%)	7 (15.6%)	6 (13.3%)	5 (11.1%)
**Ulcers**	40 (88.9%)		4 (8.9%)	1 (2.2%)
**Mucoceles**	39 (86.7%)	3 (6.7%)	3 (6.7%)	0

With respect to self-reported severity of oral cGvHD symptoms over the last 7 days, patients reported the highest scores concerning oral dryness (5.4 ± 2.9), followed by sensitivity of the oral mucosa during food and drink consumption (4.0 ± 3.1) and oral pain (2.5 ± 3.0). Patients with objectively assessed oral manifestations of mucosal cGvHD experienced more oral pain (3.7± 3.1) compared with patients in which oral manifestations of mucosal cGvHD were not observable at the time of the study assessment (1.2 ± 2.1) (Mann–Whitney *U*-test, *p* = 0.004). Patients with oral mucosal cGvHD manifestations (4.9 ± 2.9) also noticed more oral sensitivity compared with patients without oral manifestations (2.9 ± 3.1) (Mann–Whitney *U*-test, *p* = 0.012). There was no difference in the reported oral dryness between patients with and without oral mucosal cGvHD (Mann–Whitney *U*-test, *p* > 0.05).

### Taste

**Figure 1:**
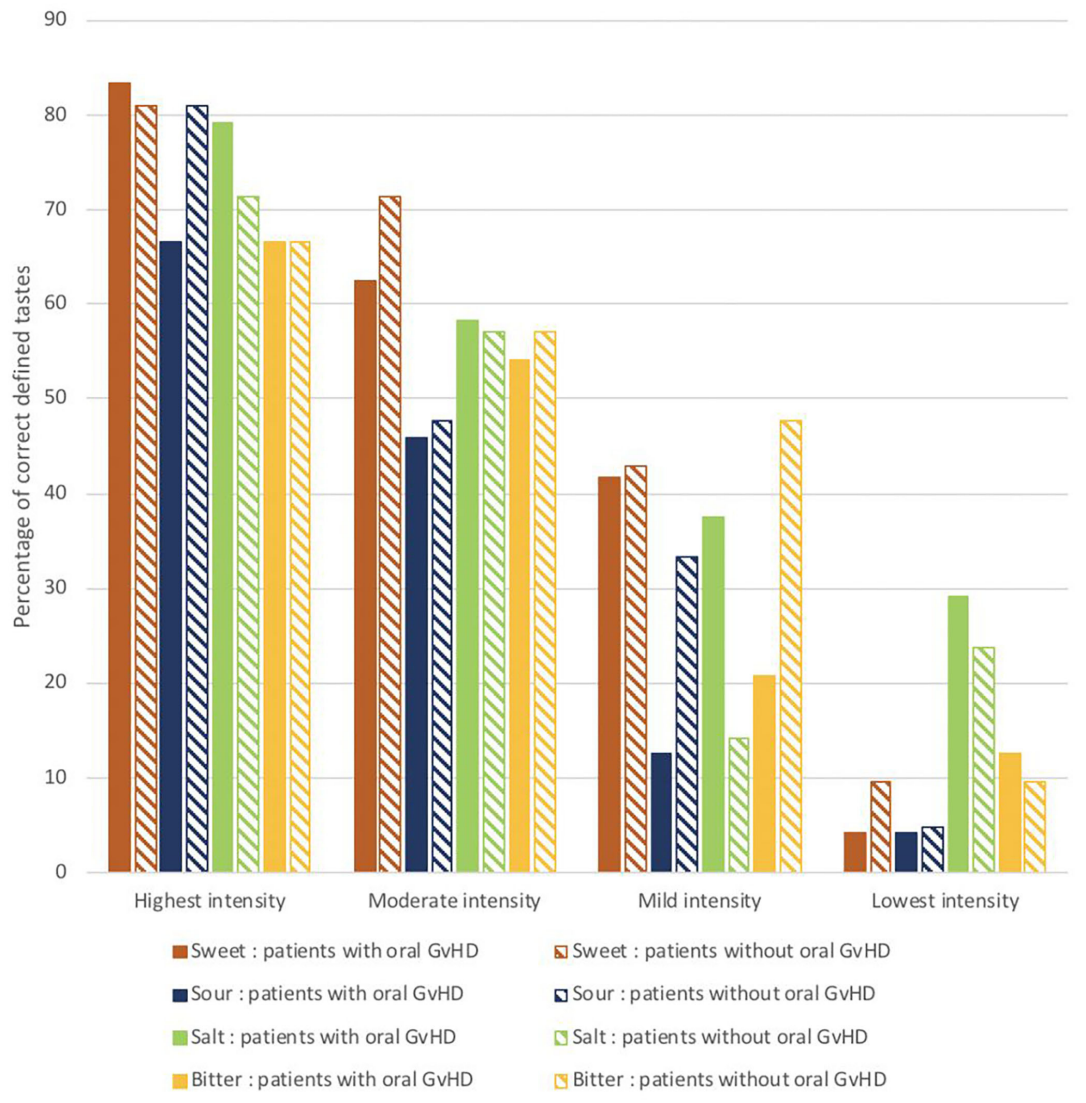
The percentages of correctly identified tastes at different test concentrations in the clinical taste evaluation test conducted in patients with oral mucosal cGVHD vs. those without oral mucosal cGVHD (*N* = 45).

A reduced ability to taste (hypogeusia) was assessed in the majority of patients (68.9%). Although most patients were able to detect all the four tastes: sweet, salt, bitter, and sour at the highest test intensity, their ability to detect tastes decreased with the reduction of the concentration on the test strips (Figure 1). In none of the patients taste ability was completely absent (ageusia).

**Figure 2:**
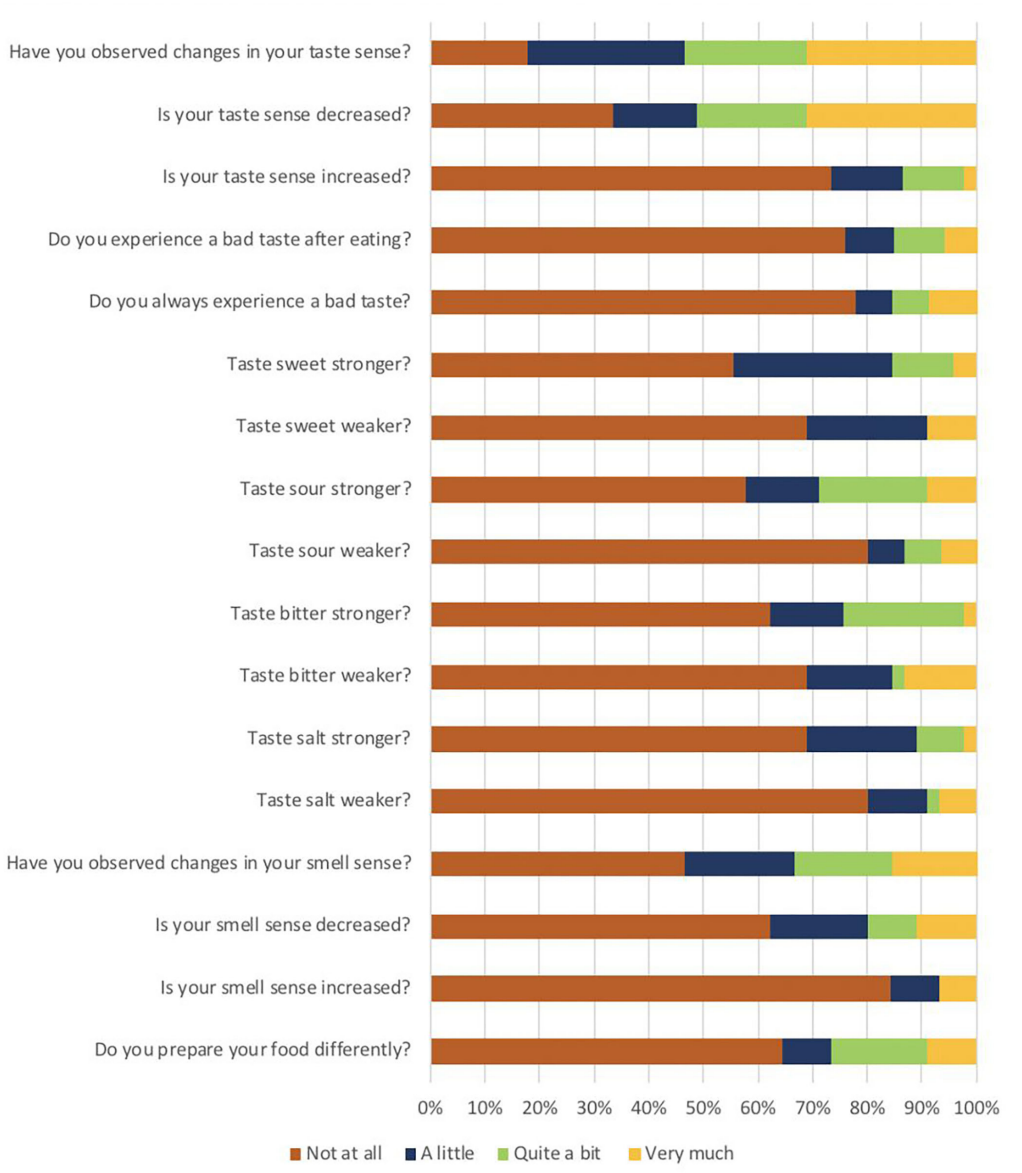
Distribution of the responses to the taste and smell addendum of the EORTC QLQ–C30 (*N* = 45).

From all the patients, 31.1% reported severe taste alterations and 22% experienced taste alterations “quite a bit”, this was most often a decrease in taste sensitivity. An increased taste sensitivity was reported by 13.3% of patients. Bitter and sour were reported as being more intensively experienced by 24–29% of patients (Figure 2).

As suggested by the discrepancy in objective and patient-reported taste outcomes, patients with hypogeusia were not always aware of their altered taste sense. Not all noticed reduced taste sense.

There was no significant difference in ability to taste when comparing patients with and without oral mucosal cGvHD (Table 3, Fisher–Freeman–Halton exact test, *p* > 0.05).

**Table 3:** Distribution of oral mucosal cGvHD and hypogeusia (objective-reduced smell ability).

		**Hypogeusia**	**Normogeusia**	**Total**	**Fisher's exact test (2-sides)**	***P*-value**
**GvHD**	**Present**	19	5	24		
	**Not present**	12	9	21	0.196	0.111
	**Total**	31	14	45		

### Smell

**Figure 3:**
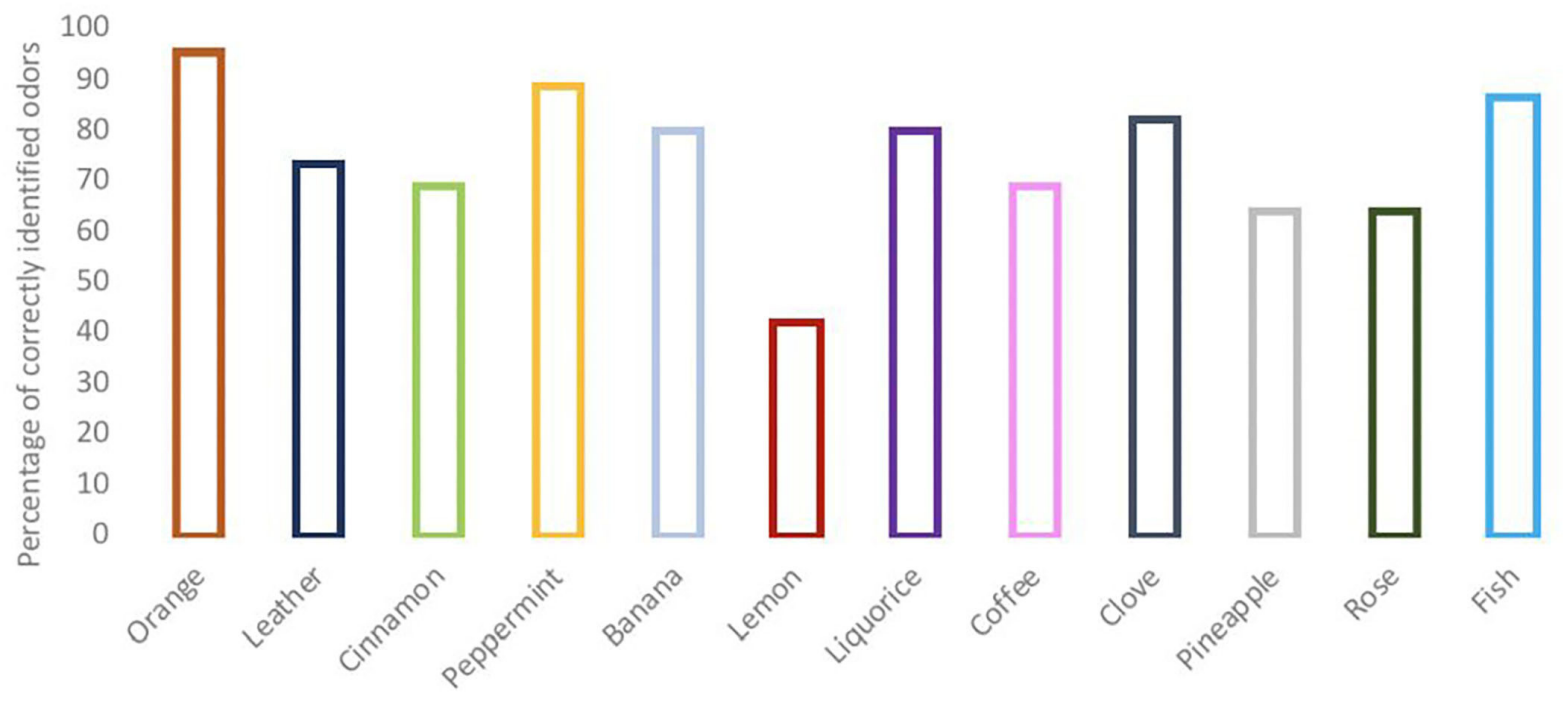
Outcomes of the clinical smell evaluation test.

Smell disturbances were found in 18 patients (40%); of which 28.9% had a reduced ability (hyposmia) and 11.1% had a complete inability (anosmia) to detect the odors tested. The most commonly correct identified odor was orange, followed by peppermint. Lemon odor was the least often identified correctly (Figure 3).

In total, 15.6% of patients reported having “very much” smell alterations. In total, 17.8% experienced that the smell has changed “quite a bit” and 20% experienced “a bit”. Part of the patients (11.1%) reported a severe overall reduction in their smell sensitivity, whereas 6.7% experienced that their ability to smell had increased “very much” (Figure 2).

Most patients with objectively assessed anosmia or hyposmia also reported a disturbance in their sense of smell. They experienced a reduced sensitivity of their smell sense (Fisher–Freeman–Halton exact test, *p* = 0.002) or an alteration in smell perception more often, compared with patients with a normal smell sense (Fisher–Freeman–Halton exact test, *p* = 0.026). There was no difference in smell sense between patients with or without manifestations of oral mucosal GvHD (Table 4, Fisher–Freeman–Halton exact test, *p* > 0.05).

**Table 4:** Relation between oral mucosal cGvHD and smell sense.

		**Anosmia**	**Hyposmia**	**Normosmia**	**Total**	**Fisher-Freeman-Halton**	***P*-value**
						**exact test**	
**GvHD**	**Present**	2	8	11	21		
	**Not present**	3	5	16	24	1.668	0.463
	**Total**	5	13	27	45		

### Sialometry and xerostomia

About 85% of patients had a normal level of (un)stimulated salivary flow. The pH of the (un-) stimulated saliva was on average slightly below the normal values (Table 5). The sensation of oral dryness (xerostomia) was reported by 75.6% of patients (EORTC QLQ–OH15), of which 15.6% reported “a bit” oral dryness, 33.3% reported “quite a bit”, and 26.7% reported “very much” oral dryness. There was no significant association between categories of (un)stimulated salivary flow and taste and smell disorders (Table 6, Fisher–Freeman–Halton exact test, *p* > 0.05).

**Table 5:** Salivary flow classification.

	**Stimulated**			**Unstimulated**		
	**N (%)**	**Mean ±SD**	**Ref. Value**	**N (%)**	**Mean ±SD**	**Ref. Value**
**Hyposalivation**	7 (15.6%)		**<0.5 ml/min**	6 (13.3%)		**<0.1 ml/min**
**Normal**	38 (84.4%)		**>0.5 ml/min**	39 (86.7%)		**>0.1 ml/min**
**pH**		6.9 ± 0.5	**7.0–8.0 pH**		6.2 ± 0.3	**6.8–7.5 pH**

**Table 6:** Distribution of taste and smell disorders and salivary flow.

	**Hyposalivation**	**Normal salivary flow**	**Total**	**Fisher-Freeman-Halton**	***P*-value**
				**exact test**	
**Unstimulated**					
**Hypogeusia**	6	25	31	-	0.156
**Normogeusia**	0	14	14		
**Total**	6	23	45		
**Anosmia**	1	4	5	0.908	0.832
**Hyposmia**	1	12	13		
**Normosmia**	4	23	27		
**Total**	6	39	45		
**Stimulated**					
**Hypogeusia**	5	26	31	–	1.000
**Normogeusia**	2	12	14		
**Total**	7	38	45		
**Anosmia**	2	3	5	2.701	0.307
**Hyposmia**	1	12	13		
**Normosmia**	4	23	27		
**Total**	7	38	45		

### Quality of life

In general, patients were moderately positive about their overall QoL at least 100 days after transplantation (EORTC QLQ–C30: 67.2 ± 24.6). However, on average patients reported a decreased OH-related QoL of 24.0 ± 16.0 (EORTC OH-15). Most reported problems included soreness in their mouth, sores in the corners of their mouth, a dry mouth, sensitivity to food and drink, taste alterations, and problems eating solid foods (Table 7). There were no differences in OH-related QoL between patients with and without taste disorders, smell disorders, or manifestations of oral mucosal cGvHD (*p* > 0.05).

**Table 7:** Differences between (oral health related) quality of life (sub)scales and taste/smell.

	**Subscales**		**Taste**				**Smell**					**cGvHD**			
		**Overall**	**Hypogeusia**	**Normogeusia**			**Anosmia**	**Hyposmia**	**Normosmia**			**Not**	**Present**		
												**present**			
		**Mean**	**Mean**	**Mean**	**Coefficient^a^**	**p-value**	**Mean**	**Mean**	**Mean**	**Coefficient^b^**	**p-value**	**Mean**	**Mean**	**Coefficient^a^**	**p-value**
		**±SD**	**±SD**	**±SD**			**±SD**	**±SD**	**±SD**			**±SD**	**±SD**		
**EORTC QLQ-C30**	Global health status/QoL^c^	67.2 ±24.6	69.6 ± 26.0	61.9 ± 21.1	163.0	0.186	63.3 ± 32.6	70.5 ± 16.5	66.4 ± 27.0	0.066	0.969	71.8 ± 20.7	63.2 ± 27.5	210.0	0.342
**EORTC** **QLQ-OH15**	Oral health-QoL^c^	24.0 ± 16.0	25.7 ± 16.7	20.2 ± 14.2	180.5	0.377	25.8 ± 18.0	21.5 ± 10.7	24.8 ± 18.1	0.237	0.888	19.6 ± 14.6	27.8 ± 16.6	182.0	0.112
	Sticky saliva^d^	22.2 ±33.3	23.7 ± 36.7	19.0 ± 25.2	215.0	0.948	20.0 ± 44.7	18.0 ± 25.9	24.7 ± 35.3	0.440	0.802	22.2 ± 33.9	22.2 ± 33.6	251.5	0.997
	Sensitivity to food and drink^d^	40.7 ±33.2	45.2 ± 35.0	31.0 ± 27.6	168.5	0.235	46.7 ± 44.7	38.5 ± 32.9	40.7 ± 32.5	0.174	0.917	41.3 ± 37.9	40.3 ± 29.5	249.0	0.943
	Sore mouth^d^	48.1 ±37.9	49.5 ± 40.3	45.2 ± 33.6	207.0	0.806	46.7 ± 50.6	43.6 ± 37.0	50.6 ± 37.4	0.339	0.884	47.6 ± 42.9	48.6 ± 34.0	242.5	0.822
**OHIP-14**	Functional limitations^d^	4.2 ± 2.3	4.4 ± 2.3	3.6 ± 2.2	178.0	0.329	5.6 ± 3.6	3.5 ± 2.0	4.3 ± 2.0	2.417	0.308	3.7 ± 2.5	4.6 ± 2.0	179.0	0.086
	Physical pain^d^	5.0 ± 2.5	5.1 ± 2.4	4.8 ± 2.6	200.0	0.681	6.0 ± 2.7	5.5 ± 2.8	4.6 ± 2.3	1.954	0.387	4.2 ± 2.0	5.7 ± 2.7	173.0	0.069
	Psychological discomfort^d^	3.1 ± 1.8	3.4 ± 2.1	2.6 ± 1.1	181.5	0.326	3.2 ± 1.8	3.2 ± 1.8	3.1 ± 1.9	0.049	0.972	2.7 ± 1.2	3.5 ± 2.2	207.0	0.249
	Physical disability^d^	4.3 ± 2.4	4.4 ± 2.6	4.1 ± 2.1	209.0	0.848	5.2 ± 3.0	4.8 ± 2.5	3.9 ± 2.3	1.842	0.408	4.1 ± 2.6	4.5 ± 2.3	215.0	0.390
	Psychological disability^d^	2.8 ± 1.2	2.9 ± 1.4	2.6 ± 0.9	197.0	0.600	2.4 ± 0.9	2.8 ± 1.1	2.9 ± 1.3	0.787	0.690	2.6 ± 1.2	3.0 ± 1.3	207.5	0.255
	Social disability^d^	2.8 ± 1.3	2.9 ± 1.5	2.6 ± 0.9	209.5	0.824	2.8 ± 1.8	2.9 ± 1.3	2.8 ± 1.4	0.589	0.767	2.4 ± 0.9	3.3 ± 1.6	170.5	0.030*
	Handicap^d^	2.9 ± 1.5	3.0 ± 1.6	2.8 ± 1.2	208.0	0.818	3.8 ± 3.0	3.2 ± 1.3	2.6 ± 1.1	1.882	0.394	2.6 ± 1.1	3.3 ± 1.7	189.5	0.106

a Mann-Whitney U-test.

b Kruskal-Wallis H-test.

* p-value is significant <0.05 level (2-tailed).

c higher scores (EORTC: max. 100, OHIP: max. 10) denote an improved QoL (lower symptom burden).

d higher scores (EORTC: max. 100, OHIP: max. 10) denote an impairment in QoL (higher symptom burden).

The complaint most often reported by using the OHIP-14 questionnaire was oral pain (Table 7). Social disability assessed by the OHIP-14 was significantly more often reported by the patients with oral mucosal GvHD compared with those without these manifestations (Mann–Whitney *U*-test, *p* = 0.030).

## Discussion

The purpose of this study was to identify the prevalence, nature, severity of taste, and smell disturbances in patients with oral cGvHD and to examine whether taste and smell disturbances are related to manifestations of oral mucosal cGvHD, salivary flow, and global or OH-related QoL.

Reduced ability to taste was identified in 68.9% of patients, although not all the patients reported having reduced taste. Reduced smell ability was less common, 40% of patients had hyposmia (28.9%) or anosmia (11.1%). Most of the patients with hyposomia/anosmia also reported having disturbed smell. The presence of taste and smell disturbances were equally divided between patients with and without manifestations of oral mucosal cGvHD, which is in accordance with the findings of others [4]. Also, no significant association could be identified between taste sense and salivary flow. Taste and smell disturbances seemed not to have a significant negative impact on patients' overall and OH-related QoL.

The prevalence of objectively assessed hypogeusia to perceive basic flavors in this study (68.9%) is in line with the 66.6% prevalence reported by Ferreira and coworkers during the neutropenic phase after HSCT [29]. Our study, in which participants were evaluated at least 100 days post-transplant, suggests that patients may suffer from hypogeusia far beyond the neutropenic phase. Patient-reported taste disturbances may fade away within 3 years after HSCT [4, 9]. Patients in this cross-sectional study experienced taste problems from 3 months up to over 10 years after transplantation. Interestingly, some patients with an objective reduced taste ability were not aware of their reduced taste, indicating that they may have adapted to having reduced taste.

Taste and smell receptor cells have a short lifespan from 7 up to 10 days, making them vulnerable to the toxic effects of the conditioning regimen consisting of chemotherapy and/or radiotherapy [30, 31]. Radiation-related taste disturbances because of the altering the taste pores structure or thinning the papilla epithelium have not been reported for doses under 20 Gy administered to the head and neck region. Patients in this study received a total body irradiation dose of 10 Gy at maximum. Therefore, the effect of radiation therapy to taste and smell disturbances in our study was likely negligible.

It is interesting to note that one of the best preserved tastes in this study was the bitter taste, which is believed to evolve for early detection of potentially poisoning substances [32]. Antineoplastic drugs, such as cyclophosphamide, could play a role by disrupting taste sensation conduction resulting in specific taste sensations without stimulating the taste receptors or requiring the presence of the corresponding flavor molecules [31, 33]. Also, commonly used medications, such as antimicrobials, corticosteroids, and psychoactive drugs, could adversely influence the sense of taste and smell, either by altering ability to taste and smell, or by producing perceptual distortions, or phantom sensations because of the neurotoxity [34]. The diversity and amount of drugs used by our patients (over a 100 types) used made it impossible to determine their impact on taste and smell.

All patients included in this study had oral manifestations of mucosal cGvHD at the time of evaluation or a history of recently having such manifestations diagnosed in our oral GvHD clinic. Oral mucosal manifestations of cGvHD may vary significantly over time (even over several weeks) as a result of multiple factors, namely, therapy-related factors (i.e., immunosuppressive, other medications) and patient-related factors (i.e., infections, stress/anxiety that may trigger GvHD, adherence to therapy). Oral mucosal cGHVD manifestations were mostly mild-to-moderate in nature. As observed by us and others, patients may still report multiple oral cGvHD-related complaints in the absence of visible manifestations [35, 36]. According to Sato and coworkers, patient-reported oral cGvHD is a significant predictive factor for taste disorders in alloHSCT recipients 3 months or more post-transplant [9].

The salivary glands may also be affected by cGvHD, resulting in hyposalivation. Changes in biochemical and immunological salivary components are associated with the reduced salivary function after alloHSCT, which may reduce the ability to taste and oral/mucosal health [37]. We did not find taste/smell disturbances to be related to hyposalivation, but prospective studies with larger numbers of patients are needed.

Scordo et al. reviewed studies directed to taste alterations following HSCT and presented potential pathobiological mechanisms [38]. Although cells and tissues crucial for taste and smell perception may be damaged by the GvHD-associated inflammation, there is no clear understanding yet of how cGvHD may be linked to taste and smell dysfunction. To shed more light on the etiology and pathobiology of taste and smell alterations, a holistic approach aiming at identifying potential cellular targets and shared mechanisms affecting multiple organs and sites of patients with cGvHD, namely, the oral and nasal epithelium, lungs, kidneys, and liver may be helpful. Moreover, recent studies on COVID-19-related dysgeusia and anosmia may also provide clues for a better mechanistic understanding. Interestingly, the renin–angiotensin system has been proposed to be a key player in the taste sensitivity modulation, warranting further investigation [39].

Dominant drivers in patients' food choice are taste and smell. However, eating is more than just the ingestion of food. Eating has an important role in cultural and social identity, religion, and family memories. As a consequence, taste/smell disorders could not only lead to malnutrition and weight loss, but also impair social interactions resulting in reduced QoL [6]. This study identified a decreased oral health related quality of life. However, we could not identify a significant difference between patients with and without taste and smell disorders using the EORTC-15 and OHIP-14. In general, patients with GvHD in this study were able to adjust their lifestyle to the limitations of their current health state and appreciate their new life after transplantation. However, they reported a negative impact of oral cGvHD on their social life. In this study, the focus was on oral cGvHD, not taking into account GvHD at other body sites or any comorbidities which may have negatively influenced overall QoL.

At present, available supportive care interventions to ameliorate taste disturbances are scarce and there is only limited evidence for their efficacy. Interventions include dietary counseling, amifostine, zinc supplementation, and photobiomodulation [31, 40, 41]. Thus, developing effective approaches for the prevention and treatment of these problems is an urgent clinical need.

Taken together, our results indicate a high prevalence of hypogeusia, whereas smell disturbances were less common but still represent a significant clinical problem. Future work is necessary to better understand the prevalence and pathogenesis of taste and smell disturbances, and their impact on patients' physical and mental well-being. Longitudinal studies are required in which significant numbers of patients stratified for age and gender, oral hygiene and disease, cancer diagnosis, cancer treatment before conditioning therapy, stem cell source, presence of any oral, or non-oral cGVHD are followed before and long term after transplant to evaluate patterns of taste and smell disturbances and potential risk factors. As the ability to taste umami was reported to be reduced, testing should include umami [9]. Structurally evaluating taste and smell ability could contribute to gaining awareness of this problem among clinicians and draw more attention toward the need of developing efficacious supportive care options tailored to the specific needs of the patients.

## Conclusion

Taste and smell disturbances are prevalent among the alloHSCT recipients even a considerable time post-transplant. Most patients reported a decreased OH-related QoL, but specific impact of taste and smell disturbances remains to be elucidated.

## Data availability statement

The raw data supporting the conclusions of this article will be made available by the authors, without undue reservation.

## Ethics statement

The studies involving human participants were reviewed and approved by NL69437.018.19. The patients/participants provided their written informed consent to participate in this study.

## Author contributions

MB contributed to conception, design, data acquisition and interpretation, performed all statistical analyses, and drafted the manuscript. JR-D contributed to conception, data acquisition and critically revised the manuscript. MH contributed to conception, design and critically revised the manuscript. FR contributed to conception, data interpretation and critically revised the manuscript. AL contributed to conception, design, contributed to analysis and data interpretation, drafted and critically revised the manuscript. All authors contributed to the article and approved the submitted version.

## Conflict of interest

The authors declare that the research was conducted in the absence of any commercial or financial relationships that could be construed as a potential conflict of interest.

## Publisher's note

All claims expressed in this article are solely those of the authors and do not necessarily represent those of their affiliated organizations, or those of the publisher, the editors and the reviewers. Any product that may be evaluated in this article, or claim that may be made by its manufacturer, is not guaranteed or endorsed by the publisher.
